# Applying polygenic risk scoring for psychiatric disorders to a large family with bipolar disorder and major depressive disorder

**DOI:** 10.1038/s42003-018-0155-y

**Published:** 2018-10-08

**Authors:** Simone de Jong, Mateus Jose Abdalla Diniz, Andiara Saloma, Ary Gadelha, Marcos L. Santoro, Vanessa K. Ota, Cristiano Noto, Major Depressive Disorder and Bipolar Disorder Working Groups of the Psychiatric Genomics Consortium, Naomi R. Wray, Stephan Ripke, Manuel Mattheisen, Maciej Trzaskowski, Enda M. Byrne, Abdel Abdellaoui, Mark J. Adams, Esben Agerbo, Tracy M. Air, Till F. M. Andlauer, Silviu-Alin Bacanu, Marie Bækvad-Hansen, Aartjan T. F. Beekman, Tim B. Bigdeli, Elisabeth B. Binder, Douglas H. R. Blackwood, Julien Bryois, Henriette N. Buttenschøn, Jonas Bybjerg-Grauholm, Na Cai, Enrique Castelao, Jane Hvarregaard Christensen, Toni-Kim Clarke, Jonathan R. I. Coleman, Lucía Colodro-Conde, Baptiste Couvy-Duchesne, Nick Craddock, Gregory E. Crawford, Gail Davies, Ian J. Deary, Franziska Degenhardt, Eske M. Derks, Nese Direk, Conor V. Dolan, Erin C. Dunn, Thalia C. Eley, Valentina Escott-Price, Farnush Farhadi Hassan Kiadeh, Hilary K. Finucane, Andreas J. Forstner, Josef Frank, Héléna A. Gaspar, Michael Gill, Fernando S. Goes, Scott D. Gordon, Jakob Grove, Christine Søholm Hansen, Thomas F. Hansen, Stefan Herms, Ian B. Hickie, Per Hoffmann, Georg Homuth, Carsten Horn, Jouke-Jan Hottenga, David M. Hougaard, Marcus Ising, Rick Jansen, Ian Jones, Lisa A Jones, Eric Jorgenson, James A. Knowles, Isaac S. Kohane, Julia Kraft, Warren W. Kretzschmar, Jesper Krogh, Zoltán Kutalik, Yihan Li, Penelope A. Lind, Donald J. MacIntyre, Dean F. MacKinnon, Robert M. Maier, Wolfgang Maier, Jonathan Marchini, Hamdi Mbarek, Patrick McGrath, Peter McGuffin, Sarah E. Medland, Divya Mehta, Christel M. Middeldorp, Evelin Mihailov, Yuri Milaneschi, Lili Milani, Francis M. Mondimore, Grant W. Montgomery, Sara Mostafavi, Niamh Mullins, Matthias Nauck, Bernard Ng, Michel G. Nivard, Dale R. Nyholt, Hogni Oskarsson, Michael J. Owen, Jodie N. Painter, Carsten Bøcker Pedersen, Marianne Giørtz Pedersen, Roseann E. Peterson, Erik Pettersson, Wouter J. Peyrot, Giorgio Pistis, Danielle Posthuma, Jorge A. Quiroz, Per Qvist, John P. Rice, Brien P. Riley, Margarita Rivera, Saira Saeed Mirza, Robert Schoevers, Eva C. Schulte, Ling Shen, Stanley I. Shyn, Engilbert Sigurdsson, Grant C. B. Sinnamon, Johannes H. Smit, Daniel J. Smith, Hreinn Stefansson, Stacy Steinberg, Fabian Streit, Jana Strohmaier, Katherine E. Tansey, Henning Teismann, Alexander Teumer, Wesley Thompson, Pippa A. Thomson, Thorgeir E. Thorgeirsson, Matthew Traylor, Jens Treutlein, Vassily Trubetskoy, André G. Uitterlinden, Daniel Umbricht, Sandra Van der Auwera, Albert M. van Hemert, Alexander Viktorin, Peter M. Visscher, Yunpeng Wang, Bradley T. Webb, Shantel Marie Weinsheimer, Jürgen Wellmann, Gonneke Willemsen, Stephanie H. Witt, Yang Wu, Hualin S. Xi, Jian Yang, Futao Zhang, Volker Arolt, Bernhard T. Baune, Klaus Berger, Dorret I. Boomsma, Sven Cichon, Udo Dannlowski, E. J. C. de Geus, J. Raymond DePaulo, Enrico Domenici, Katharina Domschke, Tõnu Esko, Hans J. Grabe, Steven P. Hamilton, Caroline Hayward, Andrew C. Heath, Kenneth S. Kendler, Stefan Kloiber, Glyn Lewis, Qingqin S. Li, Susanne Lucae, Pamela A. F. Madden, Patrik K. Magnusson, Nicholas G. Martin, Andrew M. McIntosh, Andres Metspalu, Ole Mors, Preben Bo Mortensen, Bertram Müller-Myhsok, Merete Nordentoft, Markus M. Nöthen, Michael C. O’Donovan, Sara A. Paciga, Nancy L. Pedersen, Brenda W. J. H. Penninx, Roy H. Perlis, David J. Porteous, James B. Potash, Martin Preisig, Marcella Rietschel, Catherine Schaefer, Thomas G. Schulze, Jordan W. Smoller, Kari Stefansson, Henning Tiemeier, Rudolf Uher, Henry Völzke, Myrna M. Weissman, Thomas Werge, Cathryn M. Lewis, Douglas F. Levinson, Anders D. Børglum, Patrick F. Sullivan, Sandra Meier, John Strauss, Wei Xu, John B. Vincent, Keith Matthews, Manuel Ferreira, Colm O’Dushlaine, Shaun Purcell, Soumya Raychaudhuri, Douglas M. Ruderfer, Pamela Sklar, Laura J. Scott, Matthew Flickinger, Margit Burmeister, Jun Li, Weihua Guan, Devin Absher, Robert C. Thompson, Fan Guo Meng, Alan F. Schatzberg, William E. Bunney, Jack D. Barchas, Stanley J. Watson, Richard M. Myers, Huda Akil, Michael Boehnke, Kimberly Chambert, Jennifer Moran, Edward Scolnick, Srdjan Djurovic, Ingrid Melle, Gunnar Morken, Aiden Corvin, Adebayo Anjorin, Radhika Kandaswamy, Jacob Lawrence, Alan W. McLean, Benjamin S. Pickard, Sarah E. Bergen, Vishwajit Nimgaonkar, Mikael Landén, Martin Schalling, Urban Osby, Lena Backlund, Louise Frisén, Niklas Langstrom, Eli Stahl, Amanda Dobbyn, Stéphane Jamain, Bruno Etain, Frank Bellivier, Markus Leber, Anna Maaser, Sascha B. Fischer, Céline S. Reinbold, Sarah Kittel-Schneider, Janice M. Fullerton, Lilijana Oruč, José G. Para, Fermin Mayoral, Fabio Rivas, Piotr M. Czerski, Jutta Kammerer-Ciernioch, Helmut Vedder, Margitta Borrmann-Hassenbach, Andrea Pfennig, Paul Brennan, James D. McKay, Manolis Kogevinas, Markus Schwarz, Peter R. Schofield, Thomas W. Mühleisen, Johannes Schumacher, Michael Bauer, Adam Wright, Philip B. Mitchell, Martin Hautzinger, John R. Kelsoe, Tiffany A. Greenwood, Caroline M. Nievergelt, Paul D. Shilling, Erin N. Smith, Cinnamon S. Bloss, Howard J. Edenberg, Daniel L. Koller, Elliot S. Gershon, Chunyu Liu, Judith A. Badner, William A. Scheftner, William B. Lawson, Evaristus A. Nwulia, Maria Hipolito, William Coryell, John Rice, William Byerley, Francis J. McMahon, Falk W. Lohoff, Peter P. Zandi, Pamela B. Mahon, Melvin G. McInnis, Sebastian Zöllner, Peng Zhang, Szabolcs Szelinger, David St. Clair, Sian Caesar, Katherine Gordon-Smith, Christine Fraser, Elaine K. Green, Detelina Grozeva, Marian L. Hamshere, George Kirov, Ivan Nikolov, David A. Collier, Amanda Elkin, Richard Williamson, Allan H. Young, I. Nicol Ferrier, Vihra Milanova, Martin Alda, Pablo Cervantes, Cristiana Cruceanu, Guy A. Rouleau, Gustavo Turecki, Sara Paciga, Ashley R. Winslow, Maria Grigoroiu-Serbanescu, Roel Ophoff, Rolf Adolfsson, Annelie Nordin Adolfsson, Jurgen Del-Favero, Carlos Pato, Joanna M. Biernacka, Mark A. Frye, Derek Morris, Nicholas J. Schork, Andreas Reif, Jolanta Lissowska, Joanna Hauser, Neonila Szeszenia-Dabrowska, Kevin McGhee, Emma Quinn, Valentina Moskvina, Peter A. Holmans, Anne Farmer, James L. Kennedy, Ole A. Andreassen, Morten Mattingsdal, Michael Gill, Nicholas J. Bass, Hugh Gurling, Andrew McQuillin, René Breuer, Christina Hultman, Paul Lichtenstein, Laura M. Huckins, Marion Leboyer, Mark Lathrop, John Nurnberger, Michael Steffens, Tatiana M. Foroud, Wade H. Berrettini, David W. Craig, Jianxin Shi, Charles Curtis, Stephen J. Newhouse, Hamel Patel, Lynsey S. Hall, Paul F. O`Reilly, Sintia I. Belangero, Rodrigo A. Bressan, Gerome Breen

**Affiliations:** 1 MRC Social Genetic and Developmental Psychiatry Centre, Institute of Psychiatry Psychology and Neuroscience, King’s College London, London, SE5 8AF UK; 2 National Institute of Health Research Biomedical Research Centre for Mental Health, Maudsley Hospital and Institute of Psychiatry, Psychology and Neuroscience, King’s College London, London, SE5 8AF UK; 3 Department of Psychiatry, Universidade Federal de São Paulo (UNIFESP/EPM), São Paulo, 04021-001 Brazil; 4 Pax Instituto de Psiquiatria, BR153, km 505, Villa Sul V, Aparecida de Goiânia, 74911-516 Brazil; 5 Department of Morphology and Genetics, Universidade Federal de São Paulo (UNIFESP/EPM), São Paulo, 04021-001 Brazil; 6 Department of Biostatistics and Health Informatics, Institute of Psychiatry, Psychology and Neuroscience, King’s College London, London, SE5 8AF UK; 7 Farr Institute of Health Informatics Research, UCL Institute of Health Informatics, University College London, London, NW1 2DA UK; 8 Division of Psychological Medicine and Clinical Neurosciences, MRC Centre for Neuropsychiatric Genetics and Genomics, Cardiff University, Cardiff, CF10 3AT UK; 9 Institute for Molecular Bioscience, The University of Queensland, Brisbane, QLD Australia; 10 Queensland Brain Institute, The University of Queensland, Brisbane, QLD Australia; 11 Stanley Center for Psychiatric Research, Broad Institute of MIT and Harvard, Cambridge, MA USA; 12 Department of Psychiatry and Psychotherapy, Charité - Universitätsmedizin, Berlin, Germany; 13 Analytic and Translational Genetics Unit, Massachusetts General Hospital, Boston, MA USA; 14 iSEQ, Center for Integrative Sequencing, Aarhus University, Aarhus, Denmark; 15 Department of Biomedicine, Aarhus University, Aarhus, Denmark; 16 Department of Clinical Neuroscience, Centre for Psychiatry Research, Karolinska Institutet, Stockholm, Sweden; 17 Department of Psychiatry, Psychosomatics and Psychotherapy, Center of Mental Health, University Hospital Würzburg, Würzburg, Germany; 18 iPSYCH, The Lundbeck Foundation Initiative for Integrative Psychiatric Research, Aarhus, Denmark; 19 Department of Biological Psychology & EMGO+ Institute for Health and Care Research, Vrije Universiteit Amsterdam, Amsterdam, Netherlands; 20 Division of Psychiatry, University of Edinburgh, Edinburgh, UK; 21 National Centre for Register-Based Research, Aarhus University, Aarhus, Denmark; 22 Centre for Integrated Register-based Research, Aarhus University, Aarhus, Denmark; 23 Discipline of Psychiatry, University of Adelaide, Adelaide, SA Australia; 24 Department of Translational Research in Psychiatry, Max Planck Institute of Psychiatry, Munich, Germany; 25 Munich Cluster for Systems Neurology (SyNergy), Munich, Germany; 26 Department of Psychiatry, Virginia Commonwealth University, Richmond, VA USA; 27 Department for Congenital Disorders, Center for Neonatal Screening, Statens Serum Institut, Copenhagen, Denmark; 28 Department of Psychiatry, Vrije Universiteit Medical Center and GGZ inGeest, Amsterdam, Netherlands; 29 Virginia Institute for Psychiatric & Behavioral Genetics, Virginia Commonwealth University, Richmond, VA USA; 30 Department of Psychiatry and Behavioral Sciences, Emory University School of Medicine, Atlanta, GA USA; 31 Department of Medical Epidemiology and Biostatistics, Karolinska Institutet, Stockholm, Sweden; 32 Translational Neuropsychiatry Unit, Department of Clinical Medicine, Aarhus University, Aarhus, Denmark; 33 Human Genetics, Wellcome Trust Sanger Institute, Cambridge, UK; 34 Statistical Genomics and Systems Genetics, European Bioinformatics Institute (EMBL-EBI), Cambridge, UK; 35 Department of Psychiatry, University Hospital of Lausanne, Prilly, Lausanne, Vaud Switzerland; 36 Genetics and Computational Biology, QIMR Berghofer Medical Research Institute, Brisbane, QLD Australia; 37 Centre for Advanced Imaging, The University of Queensland, Brisbane, QLD Australia; 38 Center for Genomic and Computational Biology, Duke University, Durham, NC USA; 39 Division of Medical Genetics, Department of Pediatrics, Duke University, Durham, NC USA; 40 Centre for Cognitive Ageing and Cognitive Epidemiology, University of Edinburgh, Edinburgh, UK; 41 Institute of Human Genetics, University of Bonn, Bonn, Germany; 42 Department of Genomics, Life&Brain Center, University of Bonn, Bonn, Germany; 43 Epidemiology, Erasmus MC, Rotterdam, Zuid-Holland, Netherlands; 44 Psychiatry, Dokuz Eylul University School of Medicine, Izmir, Turkey; 45 Department of Psychiatry, Massachusetts General Hospital, Boston, MA USA; 46 Psychiatric and Neurodevelopmental Genetics Unit (PNGU), Massachusetts General Hospital, Boston, MA USA; 47 Neuroscience and Mental Health, Cardiff University, Cardiff, UK; 48 Bioinformatics, University of British Columbia, Vancouver, BC Canada; 49 Department of Epidemiology, Harvard T.H. Chan School of Public Health, Boston, MA USA; 50 Department of Mathematics, Massachusetts Institute of Technology, Cambridge, MA USA; 51 Department of Psychiatry (UPK), University of Basel, Basel, Switzerland; 52 Human Genomics Research Group, Department of Biomedicine, University of Basel, Basel, Switzerland; 53 Department of Genetic Epidemiology in Psychiatry, Central Institute of Mental Health, Medical Faculty Mannheim, Heidelberg University-Mannheim, Baden-Württemberg, Germany; 54 Department of Psychiatry, Trinity College Dublin, Dublin, Ireland; 55 Department of Psychiatry & Behavioral Sciences, Johns Hopkins University, Baltimore, MD USA; 56 Bioinformatics Research Centre, Aarhus University, Aarhus, Denmark; 57 Department of Neurology, Danish Headache Centre, Rigshospitalet, Glostrup, Denmark; 58 Institute of Biological Psychiatry, Mental Health Center SctHans, Mental Health Services Capital Region of Denmark, Copenhagen, Denmark; 59 Brain and Mind Centre, University of Sydney, Sydney, NSW Australia; 60 Department of Functional Genomics, Interfaculty Institute for Genetics and Functional Genomics, University Medicine and Ernst Moritz Arndt University Greifswald, Greifswald, Mecklenburg-Vorpommern, DE Germany; 61 Roche Pharmaceutical Research and Early Development, Pharmaceutical Sciences, Roche Innovation Center Basel, FHoffmann-La Roche Ltd, Basel, Switzerland; 62 Max Planck Institute of Psychiatry, Munich, Germany; 63 Department of Psychological Medicine, University of Worcester, Worcester, UK; 64 Division of Research, Kaiser Permanente Northern California, Oakland, CA USA; 65 Psychiatry & The Behavioral Sciences, University of Southern California, Los Angeles, CA USA; 66 Department of Biomedical Informatics, Harvard Medical School, Boston, MA USA; 67 Department of Medicine, Brigham and Women’s Hospital, Boston, MA USA; 68 Informatics Program, Boston Children’s Hospital, Boston, MA USA; 69 Wellcome Trust Centre for Human Genetics, University of Oxford, Oxford, UK; 70 Department of Endocrinology at Herlev University Hospital, University of Copenhagen, Copenhagen, Denmark; 71 Institute of Social and Preventive Medicine (IUMSP), University Hospital of Lausanne, Lausanne, Vaud Switzerland; 72 Swiss Institute of Bioinformatics, Lausanne, Vaud Switzerland; 73 Mental Health, NHS, Glasgow, UK; 74 Department of Psychiatry and Psychotherapy, University of Bonn, Bonn, Germany; 75 Statistics, University of Oxford, Oxford, UK; 76 Psychiatry, Columbia University College of Physicians and Surgeons, New York, NY USA; 77 School of Psychology and Counseling, Queensland University of Technology, Brisbane, QLD Australia; 78 Child and Youth Mental Health Service, Children’s Health Queensland Hospital and Health Service, South Brisbane, QLD Australia; 79 Child Health Research Centre, University of Queensland, Brisbane, QLD Australia; 80 Estonian Genome Center, University of Tartu, Tartu, Estonia; 81 Department of Medical Genetics, University of British Columbia, Vancouver, BC Canada; 82 Department of Statistics, University of British Columbia, Vancouver, BC Canada; 83 DZHK (German Centre for Cardiovascular Research), Partner Site Greifswald, University Medicine, University Medicine Greifswald, Greifswald, Mecklenburg-Vorpommern Germany; 84 Institute of Clinical Chemistry and Laboratory Medicine, University Medicine Greifswald, Greifswald, Mecklenburg-Vorpommern Germany; 85 Institute of Health and Biomedical Innovation, Queensland University of Technology, Brisbane, QLD Australia; 86 Humus Inc, Reykjavik, Iceland; 87 Clinical Genetics, Vrije Universiteit Medical Center, Amsterdam, Netherlands; 88 Complex Trait Genetics, Vrije Universiteit Amsterdam, Amsterdam, Netherlands; 89 Solid Biosciences, Boston, MA USA; 90 Department of Psychiatry, Washington University in Saint Louis School of Medicine, Saint Louis, MO USA; 91 Department of Biochemistry and Molecular Biology II, Institute of Neurosciences, Center for Biomedical Research, University of Granada, Granada, Spain; 92 Department of Psychiatry, University of Groningen, University Medical Center Groningen, Groningen, Netherlands; 93 Department of Psychiatry and Psychotherapy, Medical Center of the University of Munich, Campus Innenstadt, Munich, Germany; 94 Institute of Psychiatric Phenomics and Genomics (IPPG), Medical Center of the University of Munich, Campus Innenstadt, Munich, Germany; 95 Behavioral Health Services, Kaiser Permanente Washington, Seattle, WA USA; 96 Department of Psychiatry, Faculty of Medicine, University of Iceland, Reykjavik, Iceland; 97 School of Medicine and Dentistry, James Cook University, Townsville, QLD Australia; 98 Institute of Health and Wellbeing, University of Glasgow, Glasgow, UK; 99 deCODE Genetics/Amgen, Reykjavik, Iceland; 100 College of Biomedical and Life Sciences, Cardiff University, Cardiff, UK; 101 Institute of Epidemiology and Social Medicine, University of Münster, Münster, Nordrhein-Westfalen Germany; 102 Institute for Community Medicine, University Medicine Greifswald, Greifswald, Mecklenburg-Vorpommern Germany; 103 Department of Psychiatry, University of California, San Diego, San Diego, CA USA; 104 KG Jebsen Centre for Psychosis Research, Norway Division of Mental Health and Addiction, Oslo University Hospital, Oslo, Norway; 105 Medical Genetics Section, CGEM, IGMM, University of Edinburgh, Edinburgh, UK; 106 Clinical Neurosciences, University of Cambridge, Cambridge, UK; 107 Internal Medicine, Erasmus MC, Rotterdam, Zuid-Holland, Netherlands; 108 Roche Pharmaceutical Research and Early Development, Neuroscience, Ophthalmology and Rare Diseases Discovery & Translational Medicine Area, Roche Innovation Center Basel, FHoffmann-La Roche Ltd, Basel, Switzerland; 109 Department of Psychiatry and Psychotherapy, University Medicine Greifswald, Greifswald, Mecklenburg-Vorpommern Germany; 110 Department of Psychiatry, Leiden University Medical Center, Leiden, Netherlands; 111 Computational Sciences Center of Emphasis, Pfizer Global Research and Development, Cambridge, MA USA; 112 Institute of Medical Genetics and Pathology, University Hospital Basel, University of Basel, Basel, Switzerland; 113 Institute of Neuroscience and Medicine (INM-1), Research Center Juelich, Juelich, Germany; 114 Department of Psychiatry, University of Münster, Münster, Nordrhein-Westfalen Germany; 115 Amsterdam Public Health Institute, Vrije Universiteit Medical Center, Amsterdam, Netherlands; 116 Centre for Integrative Biology, Università degli Studi di Trento, Trento, Trentino-Alto Adige Italy; 117 Department of Psychiatry and Psychotherapy, Medical Center, Faculty of Medicine, University of Freiburg, Freiburg, Germany; 118 Psychiatry, Kaiser Permanente Northern California, San Francisco, CA USA; 119 Medical Research Council Human Genetics Unit, Institute of Genetics and Molecular Medicine, University of Edinburgh, Edinburgh, UK; 120 Department of Psychiatry, University of Toronto, Toronto, ON Canada; 121 Centre for Addiction and Mental Health, Toronto, ON Canada; 122 Division of Psychiatry, University College London, London, UK; 123 Neuroscience Therapeutic Area, Janssen Research and Development, LLC, Titusville, NJ USA; 124 Institute of Molecular and Cell Biology, University of Tartu, Tartu, Estonia; 125 Psychosis Research Unit, Aarhus University Hospital, Risskov, Aarhus, Denmark; 126 University of Liverpool, Liverpool, UK; 127 Mental Health Center Copenhagen, Copenhagen Universtity Hospital, Copenhagen, Denmark; 128 Human Genetics and Computational Biomedicine, Pfizer Global Research and Development, Groton, CT USA; 129 Psychiatry, Harvard Medical School, Boston, MA USA; 130 Psychiatry, University of Iowa, Iowa City, IA USA; 131 Department of Psychiatry and Psychotherapy, University Medical Center Göttingen, Goettingen, Niedersachsen Germany; 132 Human Genetics Branch, NIMH Division of Intramural Research Programs, Bethesda, MD USA; 133 Faculty of Medicine, University of Iceland, Reykjavik, Iceland; 134 Child and Adolescent Psychiatry, Erasmus MC, Rotterdam, Zuid-Holland, Netherlands; 135 Psychiatry, Erasmus MC, Rotterdam, Zuid-Holland, Netherlands; 136 Department of Psychiatry, Dalhousie University, Halifax, NS Canada; 137 Division of Epidemiology, New York State Psychiatric Institute, New York, NY USA; 138 Department of Clinical Medicine, University of Copenhagen, Copenhagen, Denmark; 139 Department of Medical & Molecular Genetics, King’s College London, London, UK; 140 Psychiatry & Behavioral Sciences, Stanford University, Stanford, Ca USA; 141 Department of Genetics, University of North Carolina at Chapel Hill, Chapel Hill, NC USA; 142 Department of Psychiatry, University of North Carolina at Chapel Hill, Chapel Hill, NC USA; 143 Department of Biostatistics, Princess Margaret Cancer Centre, Toronto, ON Canada; 144 Dalla Lana School of Public Health, University of Toronto, Toronto, ON Canada; 145 Centre for Immunity, Infection and Evolution, University of Edinburgh, Edinburgh, UK; 146 Alvord Brain Tumor Center and Neurological Surgery Clinic, University of Washington Medical Center, Seattle, WA USA; 147 Department of Psychiatry, Icahn School of Medicine at Mount Sinai, New York, NY USA; 148 Department of Psychiatry, Brigham and Women’s Hospital, Boston, MA USA; 149 Department of Medicine, Psychiatry, Biomedical Informatics, Vanderbilt University Medical Center, Nashville, TN USA; 150 Department of Genetics and Genomic Sciences, Icahn School of Medicine at Mount Sinai, New York, NY USA; 151 Center for Statistical Genetics and Department of Biostatistics, University of Michigan, Ann Arbor, MI USA; 152 Molecular & Behavioral Neuroscience Institute and Department of Computational Medicine & Bioinformatics, University of Michigan, Ann Arbor, MI USA; 153 Biostatistics, University of Minnesota System, Minneapolis, MN USA; 154 HudsonAlpha Institute for Biotechnology, Huntsville, AL USA; 155 Department of Psychiatry and Human Behavior, University of California, Irvine, Irvine, CA USA; 156 Department of Psychiatry, Weill Cornell Medical College, New York, NY USA; 157 Department of Psychiatry, University of Michigan, Ann Arbor, MI USA; 158 Department of Medical Genetics, Oslo University Hospital, Oslo, Norway; 159 Department of Clinical Science, NORMENT, KG Jebsen Centre for Psychosis Research, University of Bergen, Bergen, Norway; 160 Division of Mental Health and Addiction, Oslo University Hospital, Oslo, Norway; 161 Department of Neuroscience, Faculty of Medicine, Norwegian University of Science and Technology (NTNU), Trondheim, Norway; 162 Department of Psychiatry, St. Olav’s University Hospital, Trondheim, Norway; 163 Department of Psychiatry, Berkshire Healthcare NHS Foundation Trust, Bracknell, UK; 164 Psychiatry, North East London NHS Foundation Trust, Ilford, UK; 165 Psychiatry and Human Genetics, University of Pittsburgh, Pittsburgh, PA USA; 166 Institute of Neuroscience and Physiology, University of Gothenburg, Gothenburg, Sweden; 167 Department of Molecular Medicine and Surgery, Karolinska Institutet and Center for Molecular Medicine, Karolinska University Hospital, Stockholm, Sweden; 168 Psychiatrie Translationnelle, Inserm U955, Créteil, France; 169 Faculté de Médecine, Université Paris Est, Créteil, France; 170 Département de Psychiatrie, Hôpital H. Mondor–A. Chenevier, Assistance Publique–Hôpitaux de Paris (AP-HP), Créteil, France; 171 Clinic for Psychiatry and Psychotherapy, University Hospital Cologne, Cologne, Germany; 172 Department of Biomedicine, University of Basel, Basel, Switzerland; 173 Neuroscience Research Australia, Sydney, NSW Australia; 174 School of Medical Sciences, University of New South Wales, Sydney, NSW Australia; 175 Mental Health Department, University Regional Hospital, Biomedicine Institute (IBIMA), Málaga, Spain; 176 Laboratory of Psychiatric Genetics, Department of Psychiatry, Poznan University of Medical Sciences, Poznan, Poland; 177 Psychiatric Center Nordbaden, Wiesloch, Germany; 178 Kliniken des Bezirks Oberbayern, Munich, Germany; 179 Department of Psychiatry and Psychotherapy, University Hospital Carl Gustav Carus, Technische Universität Dresden, Dresden, Germany; 180 Genetic Epidemiology Group, International Agency for Research on Cancer (IARC), Lyon, France; 181 Center for Research in Environmental Epidemiology (CREAL), Barcelona, Spain; 182 School of Psychiatry, University of New South Wales and Black Dog Institute, Sydney, NSW Australia; 183 Department of Clinical and Developmental Psychology, Institute of Psychology, University of Tubingen, Tubingen, Germany; 184 The Scripps Translational Science Institute and Scripps Health, La Jolla, CA USA; 185 Department of Biochemistry and Molecular Biology, Indiana University School of Medicine, Indianapolis, IN USA; 186 Department of Psychiatry, Indiana University School of Medicine, Indianapolis, IN USA; 187 Department of Psychiatry and Behavioral Neuroscience, University of Chicago, Chicago, IL USA; 188 Department of Human Genetics, University of Chicago, Chicago, IL USA; 189 Rush University Medical Center, Chicago, IL USA; 190 Department of Psychiatry and Behavioral Sciences, Howard University College of Medicine, Washington, DC USA; 191 Washington University School of Medicine, St. Louis, MO USA; 192 Department of Psychiatry, University of California San Francisco School of Medicine, San Francisco, CA USA; 193 Department of Psychiatry, University of Pennsylvania, Philadelphia, PA USA; 194 Department of Mental Health, Johns Hopkins University and Hospital, Baltimore, MD USA; 195 Neurogenomics, TGen, Phoenix, AZ USA; 196 Institute of Medical Sciences, Foresterhill, University of Aberdeen, Aberdeen, UK; 197 Department of Psychiatry, School of Clinical and Experimental Medicine, Birmingham University, Birmingham, UK; 198 Division of Neuroscience, Ninewells Hospital & Medical School, University of Dundee, Dundee, UK; 199 University of British Columbia (UBC) Institute of Mental Health, Vancouver, BC Canada; 200 Medical University - Sofia, Sofia, Bulgaria; 201 Department of Psychiatry, Mood Disorders Program, McGill University Health Center, Montreal, QC Canada; 202 Department of Neurology and Neurosurgery, Faculty of Medicine, McGill University, Montreal, QC Canada; 203 Montreal Neurological Institute and Hospital, Montreal, QC Canada; 204 Department of Neurology, Massachusetts General Hospital, Harvard Medical School, Charlestown, MA USA; 205 Biometric Psychiatric Genetics Research Unit, Alexandru Obregia Clinical Psychiatric Hospital, Bucharest, Romania; 206 Psychiatry, UMC Utrecht Hersencentrum Rudolf Magnus, Utrecht, Netherlands; 207 Human Genetics, University of California, Los Angeles, Los Angeles, CA USA; 208 Center for Neurobehavioral Genetics, University of California, Los Angeles, Los Angeles, CA USA; 209 Department of Clinical Sciences, Psychiatry, Umeå University Medical Faculty, Umeå, Sweden; 210 Applied Molecular Genomics Unit, VIB Department of Molecular Genetics, University of Antwerp, Antwerp, Belgium; 211 Institute for Genomic Health, SUNY Downstate Medical Center College of Medicine, Brooklyn, NY USA; 212 Department of Health Sciences Research, Mayo Clinic, Rochester, MN USA; 213 Department of Psychiatry & Psychology, Mayo Clinic, Rochester, MN USA; 214 Discipline of Biochemistry, Neuroimaging and Cognitive Genomics (NICOG) Centre, National University of Ireland, Galway, Galway Ireland; 215 Department of Translational Genomics, University of Southern California, Los Angeles, CA USA; 216 Cancer Epidemiology and Prevention, M. Sklodowska-Curie Cancer Center and Institute of Oncology, Warsaw, Poland; 217 Institute of Occupational Medicine, Lodz, Poland; 218 New South Wales Ministry of Health, Sydney, NSW Australia; 219 Bioinformatics and Biostatistics Unit, College of Medicine, Cardiff University, Cardiff, UK; 220 Campbell Family Mental Health Research Institute, Centre for Addiction and Mental Health, Toronto, ON Canada; 221 Institute of Medical Sciences, University of Toronto, Toronto, ON Canada; 222 Department of Natural Sciences, Centre for Coastal Research, University of Agder, Kristiansand, Norway; 223 McGill University and Genome Quebec Innovation Centre, Montreal, QC Canada; 224 Research Division, Federal Institute for Drugs and Medical Devices (BfArM), Bonn, Germany; 225 Division of Cancer Epidemiology and Genetics, National Cancer Institute, Bethesda, MD 20892 USA

## Abstract

Psychiatric disorders are thought to have a complex genetic pathology consisting of interplay of common and rare variation. Traditionally, pedigrees are used to shed light on the latter only, while here we discuss the application of polygenic risk scores to also highlight patterns of common genetic risk. We analyze polygenic risk scores for psychiatric disorders in a large pedigree (*n* ~ 260) in which 30% of family members suffer from major depressive disorder or bipolar disorder. Studying patterns of assortative mating and anticipation, it appears increased polygenic risk is contributed by affected individuals who married into the family, resulting in an increasing genetic risk over generations. This may explain the observation of anticipation in mood disorders, whereby onset is earlier and the severity increases over the generations of a family. Joint analyses of rare and common variation may be a powerful way to understand the familial genetics of psychiatric disorders.

## Abstract (web summary)

Simone de Jong et al. examine the balance of common and rare risk for psychiatric disorders in a large family with high incidence of Bipolar Disorder and Major Depressive Disorder. They find that increased polygenic risk over generations could be partially due to assortative mating, which may explain the observation of anticipation in mood disorders.

## Introduction

The development of polygenic risk scoring (PRS) has greatly advanced the field of psychiatric genetics. This approach allows for even sub-genome-wide significant threshold results from large genome-wide meta analyses to be leveraged to explore genetic risk in smaller studies^1^. The effect sizes at many individual single-nucleotide polymorphisms (SNPs), estimated by large genome-wide association studies (GWAS) on the disorder of interest, are used to calculate an individual level genome-wide PRS in individuals from an independent genetic dataset. The PRS based on the summary statistics of the schizophrenia (SCZ) GWAS by the Psychiatric Genomics Consortium (PGC)^2,3^ has proven to be most powerful in predicting not only SCZ^1,4^ but also other psychiatric disorders^5–7^. In addition, updated, more powerful, summary statistics from the Psychiatric Genomics Consortium from the latest GWAS for bipolar disorder (BPD) and major depressive disorder (MDD) are available via the PGC Data Access Portal (https://www.med.unc.edu/pgc/shared-methods).

Aside from increasing power in traditional case-control designs, PRS algorithms also open up new avenues for studying common variation. In this study, we consider the application of PRS within a family context. While pedigree studies have been traditionally used to explore rare genetic variation through linkage analyses, studying patterns of PRS throughout a pedigree would allow for assessment of phenomena like assortative mating and anticipation. Assortative (non-random) mating is a common phenomenon where mated pairs are more phenotypically similar for a given characteristic than would be expected by chance^8^. Results from a recent study by Nordsletten et al.^9^ show extensive assortative mating within and across psychiatric, but not physical disorders. This could explain some of the features of the genetic architecture of this category of disorders^9–11^. This includes anticipation, a phenomenon where later generations exhibit more severe symptoms at an earlier age, robustly reported (although not explained) in BPD^12^, and recently highlighted in genetic studies of MDD^13,14^.

In the current study, we aim to discuss the application of polygenic risk scoring for SCZ, MDD, and BPD to explore patterns of common risk variation within a family context. We illustrate our discussion by investigating the relationship between PRS and apparent assortative mating, and anticipation within a complex multigenerational pedigree affected with mood disorders.

## Results

### Study overview

We identified a large pedigree in Brazil, the Brazilian Bipolar Family (BBF), after examination of a 45-year-old female who presented with severe Bipolar Type 1 (BPI) disorder. She stated there were dozens of cases of mood disorders in the family, most of whom lived in a small village in a rural area of a large state north of São Paulo (see Methods for details). We conducted 308 interviews using the Portuguese version of the Structured Clinical Interview for DSM-IV Axis I Disorders (SCID-I)16 for family members over the age of 16 and the Portuguese version of Kiddie-SADS-Present and Lifetime Version (K-SADS-PL)17 for family members aged 6–16. Following diagnostic interviews, we conducted genotype analysis of all interviewees using the Illumina Infinium PsychArray-24. Polygenic risk scores (PRS) were assigned to each family member using PRS thresholds most predictive in discriminating affected from unaffected family members (see Methods).

### Affection status

The PRS thresholds were selected to optimally discriminate between affected (*n* = 78) versus unaffected (*n* = 147) family members with a higher score in affecteds for SCZ:PRS (Beta = 0.069, SE = 0.032, *Z*-ratio = 2.117, *p* = 0.035, *R*^2^ = 0.021), and BPD:PRS (Beta = 0.094, SE = 0.030, *Z*-ratio = 3.123, *p* = 0.002, *R*^2^ = 0.039). None of the PRS significantly discriminated between individuals having experienced a psychotic episode at some point in their lives (*n* = 25) versus the unaffected group (*n* = 147). Visualization of PRS in different diagnostic categories is shown in Supplementary Figure 1.

### Assortative mating

**Fig. 1:**
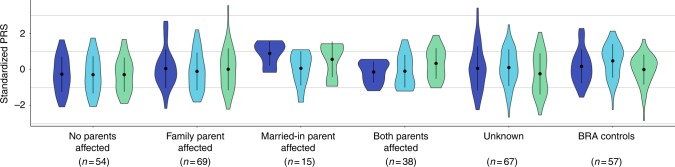
Violin plots of SCZ:PRS (dark blue plots) MDD:PRS (light blue plots) and BPD:PRS (green plots) for offspring of all spouse pair possibilities. The first category represents PRS in individuals with no affected parents, the next for individuals with an affected family member parent, followed by offspring of an affected married-in individual, and finally offspring of two affected parents. The last two sets of violin plots represent offspring of unknown spouse pairs and the BRA controls. The dot and error bars represent mean ± standard deviation of standardized PRSs

Married-in individuals were defined as individuals married to a BBF member, but having no parents in the family themselves. Of the 70 married-in individuals ascertained (irrespective of having genotype data) 19 (27%) were affected with a psychiatric disorder. This is significantly higher than the 17% population prevalence of the most common of the three disorders: MDD (Fisher’s exact *p* = 0.02)^15^. The unaffected married-in group does not differ from the general healthy population as evidenced by no significant differences in PRS as compared to the population control group (BRA; see Methods). The above led us to investigate whether we can observe assortative mating on a genetic level, using PRS. In spouse pairs, we were unable to predict the PRS of the husband, using that of his wife, even when selecting concordant (both affected or both unaffected) pairs only. We considered the possibility that the married-in individuals might confer a different genetic predisposition to mood disorders to their offspring than the original family members. The number of children contributed per spouse pair to each offspring category is shown in Supplementary Table 1. Demographics of the offspring in the different offspring categories (no affected parents (*n* = 54); one affected family member parent (*n* = 69); one affected married-in parent (*n* = 15) and two affected parents (*n* = 38)) are given in Supplementary Tables 2 and 3. Indeed, we find that offspring of an affected married-in parent show increased SCZ:PRS (Beta = 0.209, SE = 0.064, *Z*-ratio = 3.288, *p* = 0.002, *R*^2^ = 0.186, Fig. 1) and BPD:PRS (Beta = 0.172, SE = 0.066, *Z*-ratio = 2.613, *p* = 0.013, *R*^2^ = 0.126, Fig. 1) as compared to having no affected parents.

### Anticipation

**Fig. 2:**
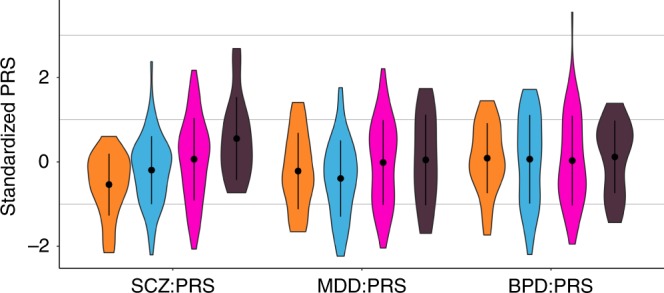
Violin plots of SCZ:PRS, MDD:PRS and BPD:PRS per generation for family members only, with results for the generations G3 (*n* = 25, orange plots), G4 (*n* = 72, light blue plots), G5 (*n* = 80, pink plots), and G6 (*n* = 16, dark purple plots) (excluding the oldest generation G2 and youngest generation G7 because of *n* = 2 sample size). The dot and error bars represent mean ± standard deviation of standardized PRSs

The BBF shows patterns of anticipation, with individuals having an earlier age at onset (AAO) in later generations. For 104 individuals (irrespective of having genotype data), the average age at onset significantly decreases over generations with G2 (*n* = 1, AAO = 8), G3 (*n* = 23, AAO = 30.2 yrs ± 21.1), G4 (*n* = 53, AAO = 31.2 yrs ± 12.3), G5 (*n* = 23, AAO = 19.7 yrs ± 9.5), and G6 (*n* = 4, AAO = 13 yrs ± 3.6) (Supplementary Figure 2) with older participants recalling their AAO directly and younger participants confirmed using clinical records or parental recall (Beta = −4.549, SE = 1.793, *Z*-ratio = −2.537, *p* = 0.013, *R*^2^ = 0.059). We hypothesized that this decrease in AAO would be reflected in a negative correlation with PRS, subsequently resulting in a pattern of increased PRS over generations. Because of a limited sample size of affected individuals per generation, a direct correlation of AAO and PRS does not reach significance, although the youngest generation (G5) does show trends towards negative correlations for SCZ:PRS and MDD:PRS (Supplementary Figure 3). The SCZ:PRS does show a significant increase over generations (Fig. 2) where *n* = 197 family members were included (46 married-in individuals were excluded from the analysis to capture inheritance patterns of SCZ:PRS) in a linear regression with generation as independent variable (Beta = 0.131, SE = 0.049, *Z*-ratio = 2.668, *p* = 0.008, *R*^2^ = 0.025). The presence of such an effect when comparing generations suggests ascertainment effects such as relying on the recall of older family member with very long duration of illness in previous generations may be masking an overall effect across the entire family.

### Balance of common and rare genetic risk

Transmission disequilibrium test analysis within the chr2p23 linkage region resulted in identification of rs1862975, a SNP originally typed on the Affymetrix linkage array (combined test *p* = 0.003). The homozygous T genotype was detected in 68% affected family members, 57% affected married-ins, 36% unaffected family members and 24% unaffected married-ins. Since this SNP was present only on the Affymetrix array, we identified rs12996218 as a proxy in CEU/TSI populations (*D*′ = 1.0, *R*^2^ = 0.92) via the LDproxy option in LDlink (Machiela et al.^16^, https://analysistools.nci.nih.gov/LDlink/). Of the 57 BRA controls, 9 individuals (15%) carried the GG genotype equivalent to the rs1862975 TT risk genotype. The distribution of the rs1862975 genotypes in affected and unaffected individuals over generations is given in Supplementary Figure 4. The number of individuals carrying the TT does not significantly change over generations in either group. None of the PRS showed a significant difference when comparing PRS for rs1862975 genotypes in affected and unaffected individuals (Supplementary Figure 5).

## Discussion

The current study is one of the first the first to probe patterns of common genetic variation within a traditional pedigree design. While increased polygenic scores in patients as compared to unaffected family members have been demonstrated recently^17^, we aimed to illustrate the possibilities of this approach by investigating apparent assortative mating and anticipation in a large multigenerational pedigree affected with mood disorders through polygenic risk scores for SCZ^2^, MDD^18^, and BPD^19^, and thereby improve mechanistic understanding of common genetic risk for psychiatric disorders.

Highlighting the possibilities of PRS applications within a family context, we set out to utilize patterns of common variation to illuminate phenomena within the family that are out of reach from traditional case/control studies. Assortative mating is one of the features in this family, where many married-in individuals are more affected with a mood disorder than the general population. As opposed to the family members, the married-in individuals were more often affected with (r)MDD instead of BP. As diagnoses were determined after the couples were married, we cannot rule out that this could be a result from a causal effect of a spouse’s mental health on that of their partner. However, non-random mating patterns have been reported in the population regarding body type, socio-economic factors and psychiatric traits^9,10^. The BBF provides a unique opportunity to look at the genetic correlation between spouse pairs and the contribution of married-in individuals to overall psychiatric morbidity. A recent study has found genetic evidence for assortative mating when studying BMI and height in spouse pairs^11^. In the BBF; the affected married-in individuals have a higher, though non-significant, polygenic score than affected or unaffected family members but it appears that we observe significant consequences of this in that the offspring of an affected married-in parent collectively show significantly increased SCZ:PRS and BPD:PRS. However, it is puzzling we do not see an effect on offspring of two affected parents (which would include a married-in parent), which could indicate this finding to be of limited statistical robustness.

A contribution of the married-in parents to a genetic driven anticipation in age of onset is supported by the increase in SCZ:PRS over generations, although our cross sectional study dataset was less well powered to find an association with age at onset within affected family members. We did observe a trend for association between age at onset and PRS in the youngest generation in this study but not when combining sample across generations. Age at onset can be considered a proxy for severity^20,21^ and has been previously associated with genetic risk in MDD^13,14^. However, this variable needs to be interpreted with caution, especially when analyzing patterns over time since it is dependent on context and memory^22^. Ascertainment bias can be a confounding factor in studies of psychiatric traits, with older generations having less access to psychiatric care and possibly misremembering the onset or nature of their first episode. In addition, although currently classified as “unaffected” or “unknown”, members of the youngest generations can still develop a psychiatric disorder in the future.

Finally, we explored the balance of common and rare risk variation through combining our current PRS results with previously performed linkage analyses. We did not find a decrease in potential rare risk allele genotypes over generations contrasting the increase in SCZ:PRS, and PRS profiles for individuals carrying rare risk genotypes are not significantly different. This indicates that these factors separately confer independent disease risk. We recognize the limitations in sample size of our pedigree and therefore the power to draw statistically robust conclusions, especially in the offspring and combined linkage and PRS analyses. Even though the BBF might not be sufficiently powered, our point is to use this dataset to illustrate our approach and emphasize the unique nature of the family enabling the study of patterns of PRS and the balance of common and rare genetic risk for psychiatric disorders conferred within families. We encourage replication in similar pedigrees including affected married-in individuals when available to fully utilize the potential of PRS in this setting.

In conclusion, our study is an exploration of PRS as a tool for investigating patterns of common genetic risk in a traditional pedigree context. The SCZ and BPD scores appear best suited in our data for teasing apart patterns of assortative mating and anticipation, whereby increased polygenic risk for psychiatric disorders is contributed by affected individuals who married into the family, adding to the already present rare risk variation passed on by the early generations^23^.

## Methods

### Subject description

The Brazilian bipolar family (BBF) was ascertained via a 45-year-old female proband who presented with severe Bipolar Type 1 (BPI) disorder and stated there were dozens of cases of mood disorders in the family, most of whom lived in a small village in a rural area of a large state north of São Paulo. Cooperation from the family and a 2003 self-published book about their history was invaluable for our ascertainment. Historically, the entire BBF consists of 960 members. Living family members > 16 years of age underwent semi-structured interviews, using the Portuguese version of the Structured Clinical Interview for DSM-IV Axis I Disorders (SCID-I)^24^. Members aged 6–16 were assessed using the Portuguese version of Kiddie-SADS-Present and Lifetime Version (K-SADS-PL)^25^. In total 308 interviews were completed, and 5 eligible members declined an interview. In the rare event of discrepancies, two independent psychiatrists reviewed them and a final consensus diagnosis was assigned. All affected and unaffected adult family members that have been included in the genetic study have given informed consent. Minors have given assent, followed by consulted consent by their parents in accordance with accepted practice in both the U.K. and Brazil. The project was approved by the Brazilian National Ethics Committee (CONEP). Table 1 contains the demographics of the subjects used in the current analysis (*n* = 243 passed genotype quality control procedures described below). The population control dataset (BRA controls) was collected in Sao Paulo, Brazil, as a control dataset in a genetic study of first-episode psychosis^26^. They were volunteers who had no abnormal psychiatric diagnoses (SCID) or family history of psychotic illness. The Research Ethics Committee of Federal University of Sao Paulo (UNIFESP) approved the research protocol, and all participants gave informed consent (CEP No. 0603/10). Demographics for *n* = 57 BRA controls can be found in Table 1.

**Table 1:** Demographics of the Brazilian bipolar family members and the Brazilian population control dataset (BRA controls) in the current study

Diagnosis	*n*	Male, female	Age (±sd)	Age of onset (±sd)	Married-in	Psychosis
BPI	17	6, 11	50.4 (±18.9)	24.9 (±14.6)	0	13
BPII	11	4, 7	38.7 (±15.2)	24.2 (±13.8)	1	4
BPNOS	8	6, 2	29.6 (±19.9)	17.0 (±18.7)	0	1
rMDD	17	5, 12	50.2 (±16.7)	27.3 (±14.1)	3	4
MDD	21	11, 10	43.8 (±17.8)	34.5 (±15.5)	6	1
SADB	1	0, 1	73	44	0	1
Schizophrenia	1	1, 0	44	36	0	1
Cyclothymia	1	0, 1	40	25	0	0
Dysthymia	1	0, 1	52	—	1	0
Unaffected	147	89, 58	36.8 (±20.0)	—	35	0
Unknown	18	14, 4	5.7 (±7.1)	—	0	—
Total	243	136, 107	37.3 (±21.0)	28.3 (±15.5)	46	25
BRA controls	57	33, 24	27.1 (±7.2)	—	—	—

The first column contains the number of individuals affected with the disorder. A breakdown of gender, age, age at onset (with ± sd; standard deviation) is given in the next columns. The married-in column contains the number of individuals in each diagnostic category married-in to the family. The last column contains counts of individuals in each category who have experienced a psychotic episode during their lifetime

Diagnostic categories are *BP1* bipolar I, *BPII* bipolar II, *BPNOS* bipolar not otherwise specified, *rMDD* recurrent major depressive disorder, *MDD* major depressive disorder, *SADB* schizoaffective disorder, schizophrenia, cyclothymia and dysthymia

### Genotype data

Following diagnostic interview, interviewers obtained whole blood in EDTA containing monovettes for adults and lesser amounts or saliva given personal preference or age (DNA Genotek Inc., Ontario, Canada). Genomic DNA was isolated from whole blood and saliva at UNIFESP using standard procedures. Whole-genome genotype data was generated using the Illumina Infinium PsychArray-24 (http://www.illumina.com/products/psycharray.html) for both the BBF and the BRA control dataset at the in-house BRC BioResource Illumina core lab according to manufacturers protocol. Samples were excluded when average call rate was <98%, missingness >1% with additional check for excess heterozygosity, sex, family relationships and concordance rates with previous genotyping assays. SNPs were excluded when missingness > 1%, MAF < 0.01 or HWE < 0.00001 and if showing Mendelian errors for the BBF dataset in Plink v1.07^27^ and v1.9^28^ or Merlin v1.1.2^29^. The BBF and BRA control datasets were QC’d separately and then merged, applying the same SNP QC thresholds to the merged dataset as well. This quality control procedure resulted in a dataset of 225,235 SNPs for 243 BBF individuals (197 family members and 46 married-in individuals) and 57 BRA controls. Eigensoft v4.2^30^ was used to check for population differences between the BBF family members, married-in individuals and BRA control sets. The BBF members self-reported mixed Southern European ancestry, confirmed by genome-wide principal components analysis showing that family members clustered closely with the Northern and Western European and Tuscan Italian populations in Hapmap3, with a relative lack of African or Native American ancestry (Supplementary Figure 6). The principal components appear to represent within-family structure, with most PCs seemingly separating subfamilies (Supplementary Figures 7 and 8). PRS analyses as described below were also performed to include subfamily as a fixed effect, controlling for household effects (Supplementary Table 3). PC1 and PC2 are significantly correlated to the SCZ:PRS (PC1 *r* = −0.131, *p* = 0.023; PC2 *r* = −0.268, *p* = 2.611 × 10^−6^), PC1 to MDD:PRS (PC1 *r* = −0.251, *p* = 1.114 × 10^−5^), and PC1 and PC2 to BPD:PRS (PC1 *r* = 0.189, *p* = 9.710 × 10^−4^; PC2 *r* = −0.123, *p* = 0.033). The principal components were not used in subsequent analyses.

### Polygenic risk scores

Polygenic risk scores for each family member (*n* = 243) and population control (*n* = 57) were generated in the same run using the PRSice v1.25 software^31^ with the publically available PGC schizophrenia GWAS^2^ as a base dataset (36,989 SCZ cases, 113,075 controls), in addition to MDD (51,865 MDD cases, 112,200 controls, not including 23andme individuals) and BPD (20,352 BPD cases, 31,358 controls) summary statistics from the latest PGC meta analyses (unpublished data^18,19^). We performed *p*-value-informed clumping on the genotype data with a cut-off of *r*^2^ = 0.25 within a 200-kb window, excluding the MHC region on chromosome 6 because of its complex linkage disequilibrium structure. Acknowledging the possibility of over-fitting, we selected the PRS thresholds most predictive in discriminating affected from unaffected family members through linear regression in PRSice for SCZ:PRS (*p* < 0.00055, 1218 SNPs), MDD:PRS (*p* < 0.0165, 715 SNPs) and BPD:PRS (*p* < 0.00005, 143 SNPs). PRS showed low to modest correlations (no covariates) amongst each other in our data (SCZ:PRS versus MDD:PRS *r* = 0.176, *p* = 0.002, SCZ:PRS versus BPD:PRS *r* = 0.124, *p* = 0.032, MDD:PRS versus BPD:PRS *r* = −0.026, *p* = 0.660).

### Linkage analysis

The main linkage analyses identifying rare genetic risk variation were performed as part of a previous paper on the BBF^23^ using the Affymetrix 10k linkage genotyping array. In order to explore the balance between common and rare risk variation, we selected the strongest signal for affected versus unaffected family members on chr2p23 (chr2:30000001-36600000, LOD = 3.83). Following the strategy described by Rioux et al.^32^, we performed a transmission disequilibrium test on the 25 markers in this linkage region in an attempt identify “linkage positive” individuals in *n* = 300 family members with one or both types of genotype array data. *N* = 155 individuals overlap with the current study and based on exploration of patterns of PRS in the current study we attempted to answer two questions: (1) with an increase of common risk variation, does rare risk variation become less important over generations, (2) do linkage positive individuals carrying the presumed risk allele show differences in PRS.

### Statistical testing

All PRS were standardized mean = 0 and SD = 1. Linear mixed model analyses were selected to be able to model covariates and relatedness within this complicated dataset. The analyses were performed using the Wald conditional F-test^33^ in ASReml-R software^34^ with one of the categories of mood disorders or family status as dependent variable and PRS as the independent variable (Supplementary Methods). Age (except for the generation analysis) and sex were fitted as fixed effects in the models. For 7 individuals in the BBF age at collection was missing and imputed to be the mean age of the relevant generation. To account for relatedness in within-family comparisons, an additive genetic relationship matrix was fitted as a random effect. The relationship matrix was constructed using LDAK software^35^ with weighted predictors and LD correction parameters suited for pedigree data, resulting in pairwise relatedness estimates and inbreeding coefficients on the diagonal. The variance explained by each PRS was calculated using: (var(*x* × *β*))/var(*y*), where *x* was the standardized PRS, *β* was the corresponding regression coefficient, and *y* was the phenotype^36^. For the analysis of offspring, we defined four spouse pair categories (“both unaffected”, “married-in parent affected”, “family parent affected”, “both affected”). While most spouse pairs contribute 1 or 2 children to the same offspring category (Supplementary Table 1); two “both affected” spouse pairs contribute 7 and 8 children, respectively. To prevent bias in our analysis in the event of more than one child per couple, we calculated the mean PRS for all offspring per spouse pair and entered this in the model as being one representative child for that couple. All *p*-values reported are uncorrected for multiple testing, since all tests concern overlapping individuals and thus have a complex dependence structure. However, we have performed 42 tests as listed in Supplementary Table 4, and so a conservative Bonferroni threshold for *p* < 0.05 is 0.001.

## Electronic supplementary material

Supplemental materials

## Data Availability

In order to ensure privacy of the family members and to comply with Brazilian regulations, restrictions apply on availability of the data as determined by the Brazilian National Ethics Committee (CONEP). Data are available upon reasonable request from the corresponding author, pending approval by the BBF ethics committee (CONEP).
