# Supplementary material for: Applying polygenic risk scoring for psychiatric disorders to a large family with bipolar disorder and major depressive disorder
Source: Commun Biol. 2018 Oct 8;1:163. doi: 10.1038/s42003-018-0155-y (PMC6175827; doi:10.1038/s42003-018-0155-y)
Supplement: Supplementary file 1 — Supplemental materials [file 42003_2018_155_MOESM1_ESM.pdf]

SUPPLEMENTARY FIGURES

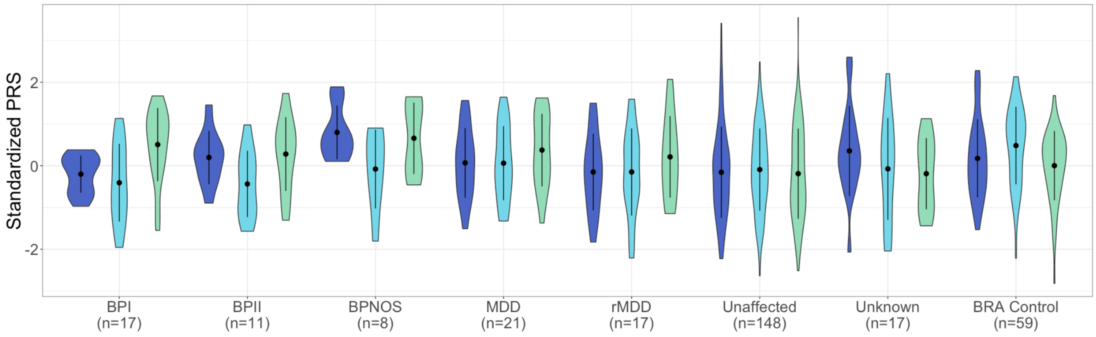

Supplementary Figure 1

Violin plots of SCZ:PRS (based on 1,218 SNPs, dark blue plots) MDD:PRS (based on 715 SNPs, light blue plots) and BPD:PRS (based on 143 SNPs, green plots) over diagnostic categories. The first three boxes represent individuals suffering from bipolar disorder I, II or NOS. The next two boxes individuals suffering from MDD or recurrent MDD, followed by the unaffected and unknown individuals and the healthy Brazilian controls. The dot and error bars represent mean +/- standard deviation of standardized PRSs.

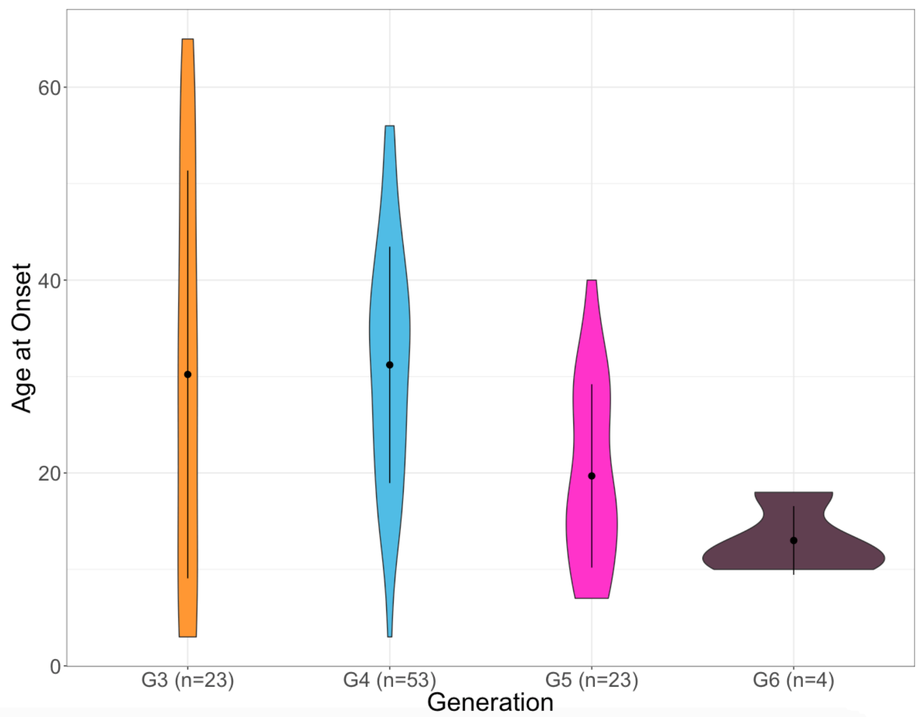

Supplementary Figure 2

Violin plot with generation on the x-axis and the age at onset on the y-axis for affected family members for Generation 3 (orange), 4 (light blue), 5 (pink) and the second-youngest generation 6 (dark purple). The oldest generation G2 and youngest generation G7 were omitted due to small (n=2) sample size. The dot and error bars represent mean +/- standard deviation of standardized PRSs. All family members have been included in the study around the same time, therefore this is reflected in the age distribution per generation of genotyped individuals irrespective of affection status with G2 (n=2, age=93.5±12.0), G3 (n=36, age=67.9yrs±9.5), G4 (n=101, age=44.5yrs±8.5), G5 (n=84, age=20.3yrs±10.0), G6 (n=18, age=10.3yrs±7.0) and G7 (n=2, age=1.5yrs±0.7).

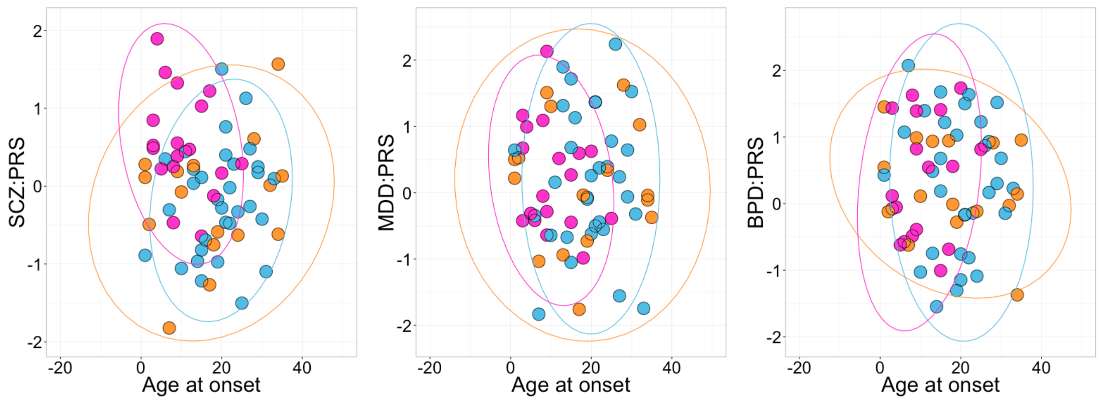

Supplementary Figure 3

The age at onset on the x-axis and standardized SCZ:PRS, MDD:PRS and BPD:PRS respectively on the y-axis for affected family members only. Individuals are colored by generation (G3 in orange, G4 in blue, and G5 in pink). Data ellipses represent 95% confidence intervals for each generation. (excluding the oldest generation G2 and youngest generations G6 and G7 because small number of affected members in these generations).

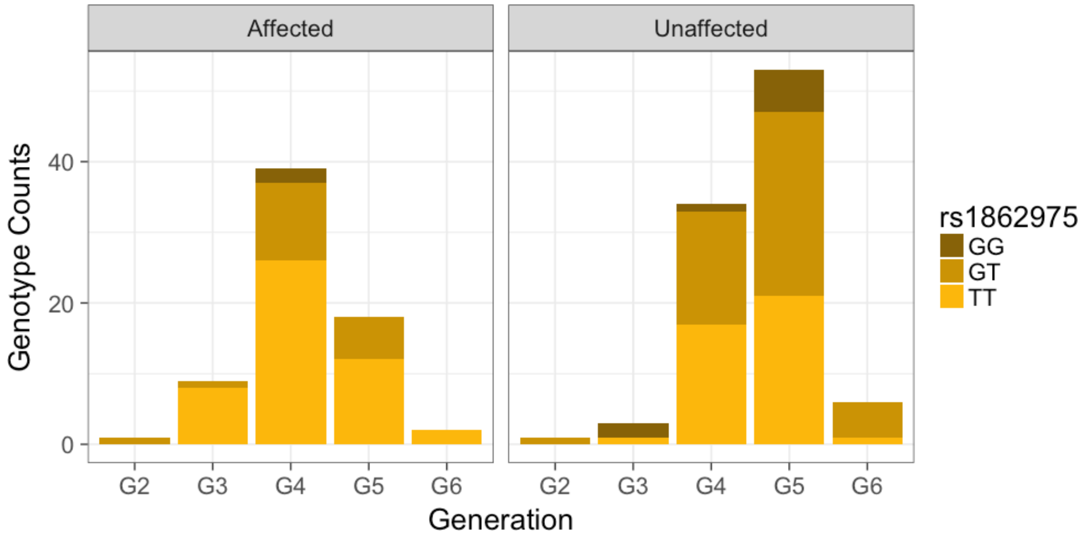

Supplementary Figure 4

Stacked barplots showing the genotype distributions of the linkage risk SNP over five generations for affected and unaffected family members (excluding married-in individuals). The left plot shows the genotype distribution of rs1862975 in n=69 affected family members, and n=97 unaffected family members. Statistical testing excluded G2 and G6 because of limited number of individuals with genotypic data available.

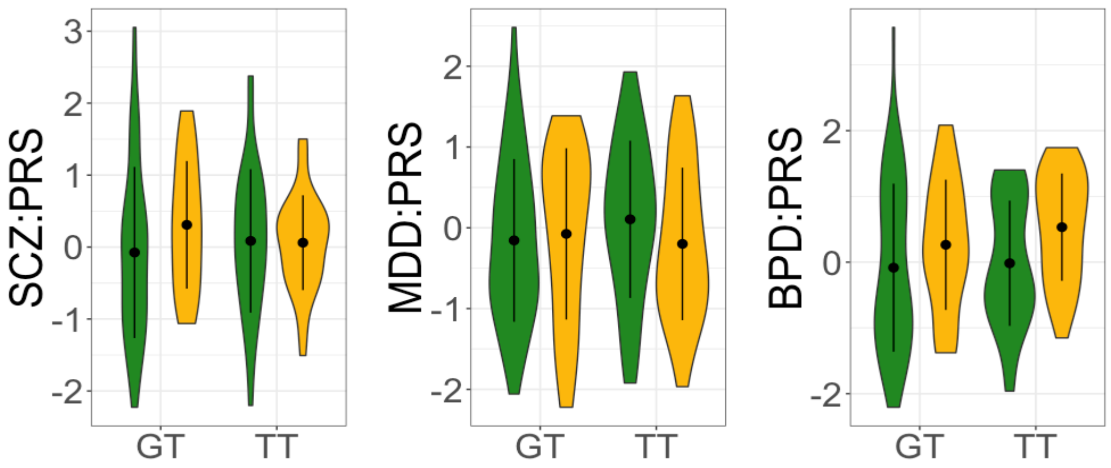

Supplementary Figure 5

Violin plots of PRS for groups carrying different linkage risk SNP genotypes in the overlapping dataset of n=60 affected (displayed in orange) and n=100 unaffected individuals (displayed in green). As only n=2 affected individuals carry the GG genotype, only GT (n=21 affected and n=54 unaffected and TT (n=37 affected and n=32 unaffected individuals) genotypes are displayed and included in statistical tests.

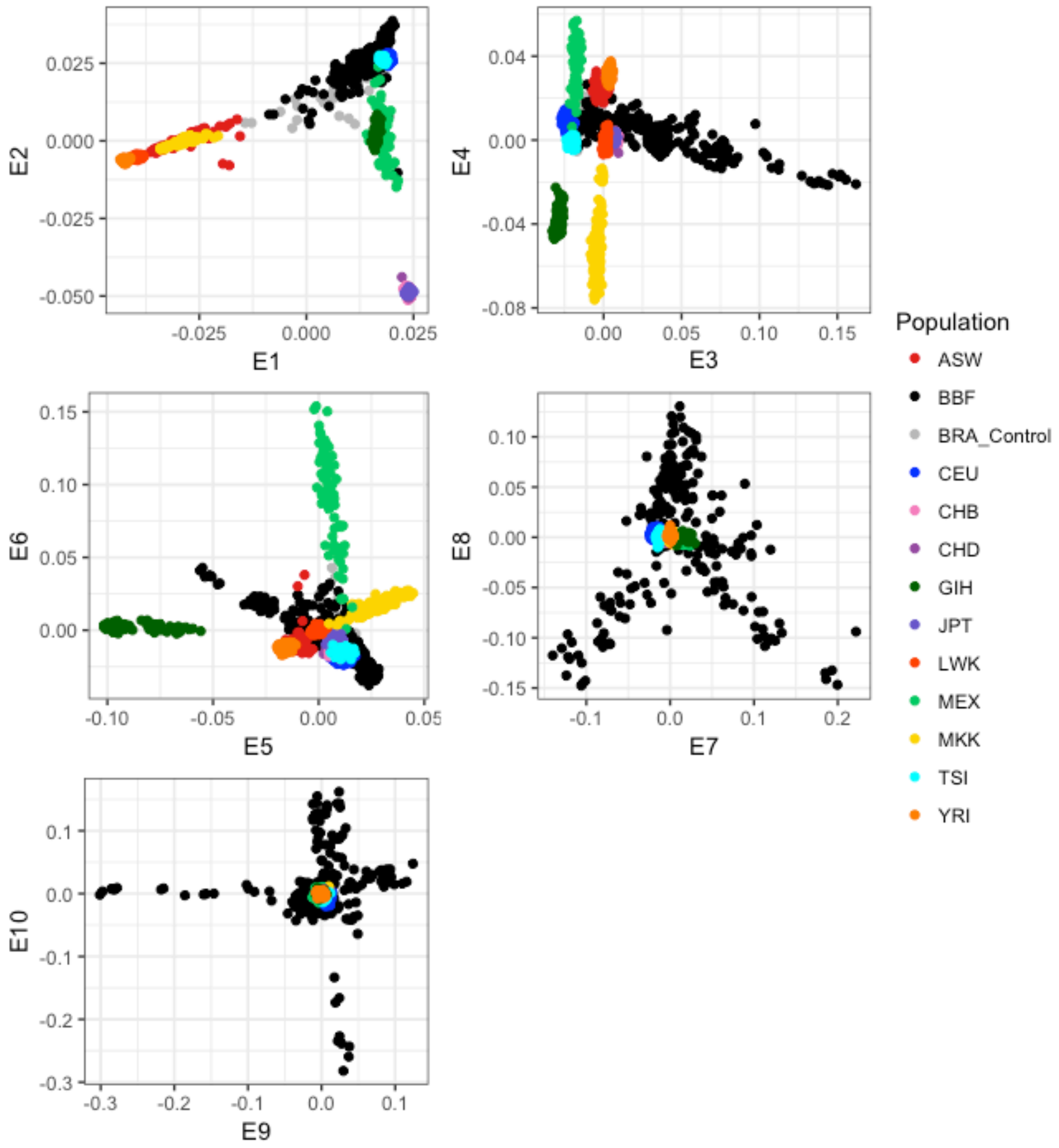

Supplementary Figure 6

Scatterplots of the first 10 principal components colored by Hapmap3 populations, BBF family members (black) and Brazilian population controls (grey). The BBF and BRA controls cluster together with European CEU and TSI populations, while showing some variation towards African populations when looking at the first two principal components.

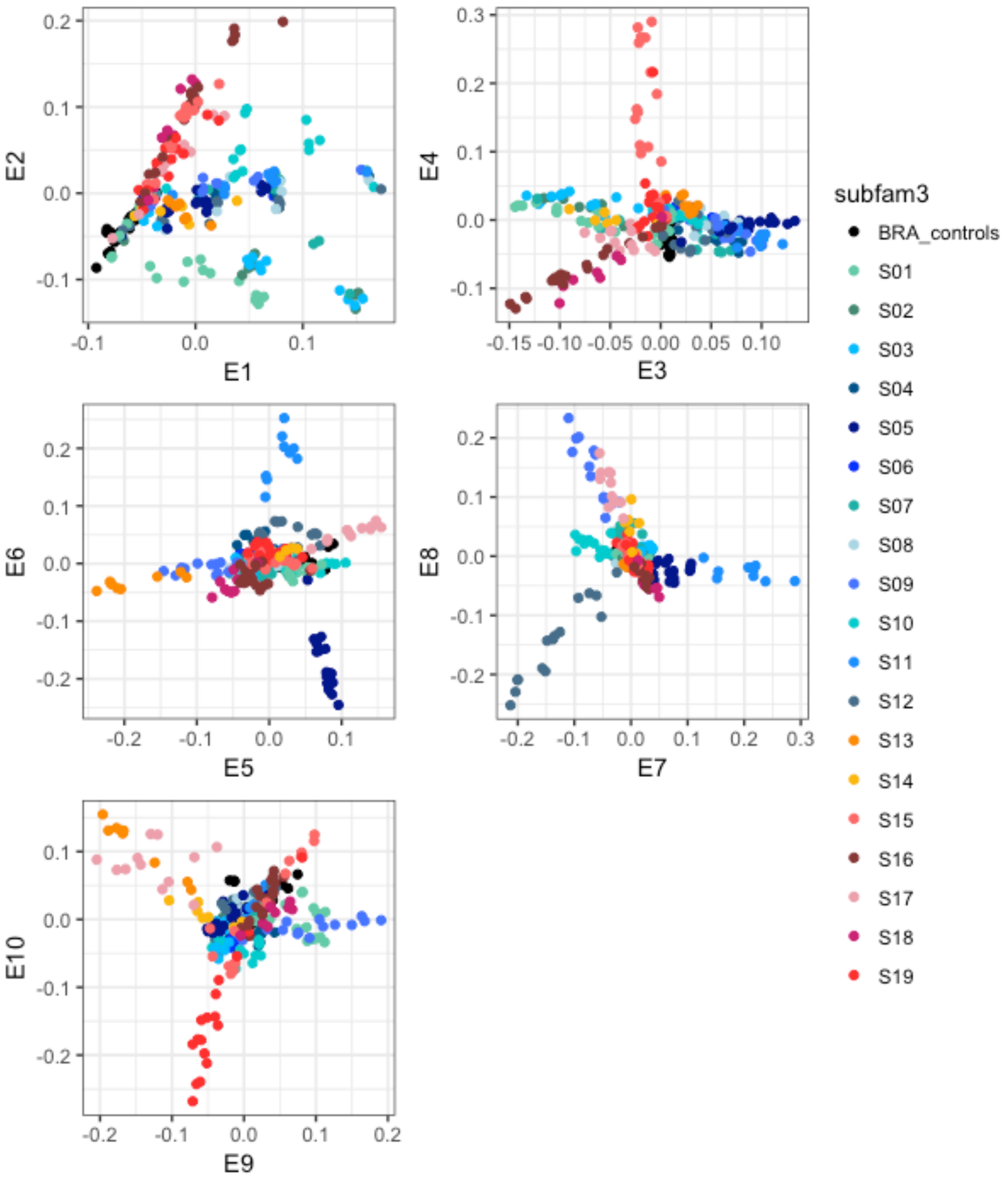

Supplementary Figure 7

Scatterplots of the first 10 principal components colored by the 19 Brazilian Bipolar family subfamilies and the Brazilian population controls in black. Subfamily 1-12 (in blue hues) belong to Branch 1, subfamily 13 and 14 (orange hues) to Branch 2 and subfamily 15-19 (red hues) to Branch 3. Principal components seemingly group together subfamilies within the BBF.

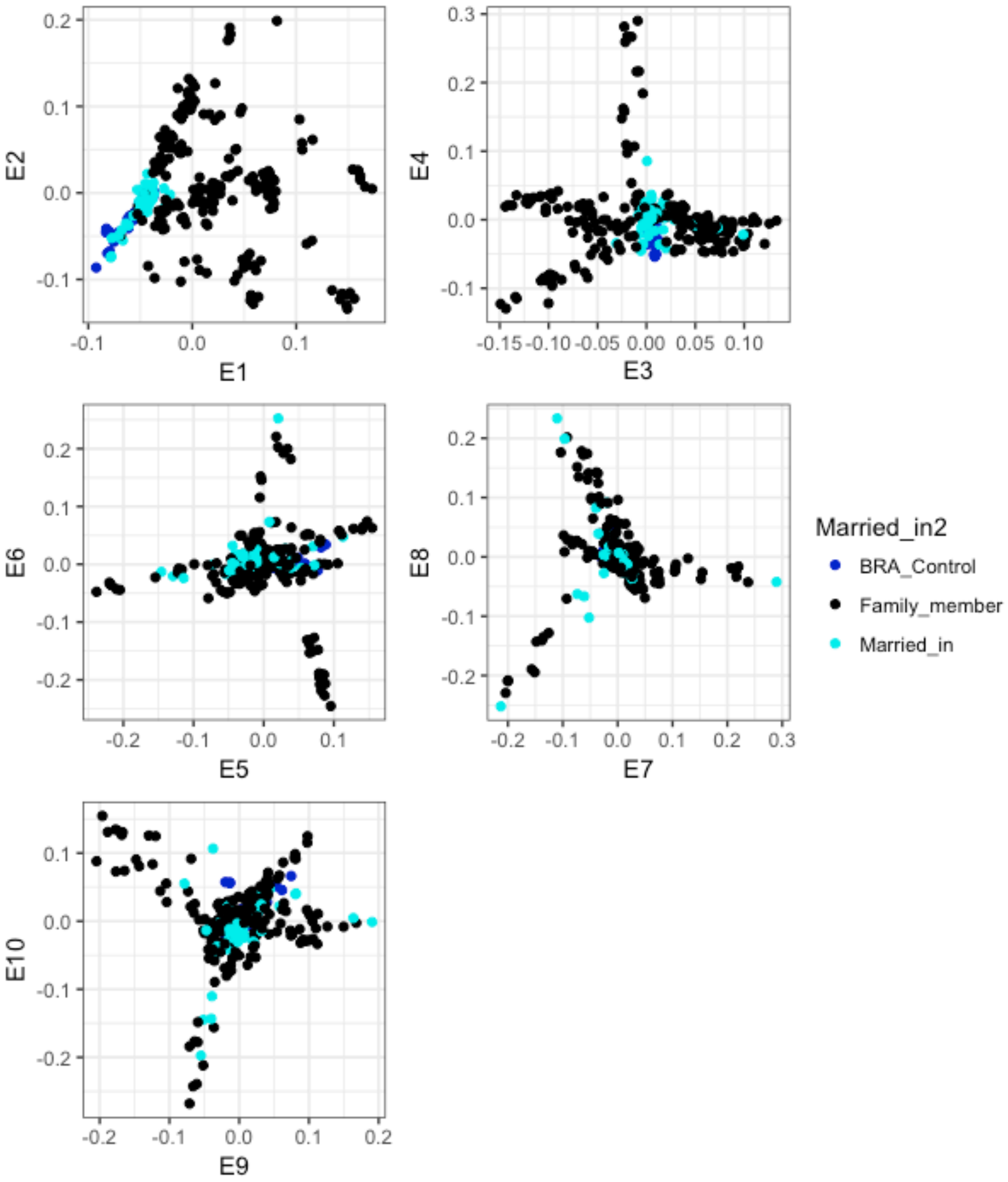

Supplementary Figure 8

Scatterplots of the first 10 principal components colored by the Brazilian Bipolar Family members in black, married-ins in light blue and Brazilian controls in dark blue.

SUPPLEMENTARY TABLES

Supplementary Table 1

Number of children contributed per spouse pair to each offspring category.

Number of children contributed per spouse pair	0_aff	1_InFam_aff	L_MarriedIn_af	2_aff	Unknown	BRA Controls
**0**	80	71	104	98	99	112
**1**	17	26	5	7	7	0
**2**	13	10	2	2	4	0
**3**	1	2	2	4	2	0
**4**	2	3	0	0	0	0
**5**	0	1	0	0	0	0
**6**	0	0	0	0	0	0
**7**	0	0	0	1	0	0
**8**	0	0	0	1	0	0

Supplementary Table 2

Demographics of the offspring of different spouse pairs (no affected parents, 1 affected family member parent, 1 affected married-in parent and offspring of two affected parents). A breakdown of gender, age, age of onset is given in the next columns.

Parental spousepair	n	Male, Female	Age (±sd)	Affected, Unaffected, Unknown	Age of onset (±sd)
**0 Affected**	54	39, 15	28.6 (±21.6)	8, 36, 10	28.7 (±16.4)
**1 In Fam affected**	69	41, 28	31.0 (±18.6)	23, 39, 7	26.4 (±14.0)
**1 Married-in affected**	15	4, 11	25.7 (±10.5)	7, 7, 1	18.0 (±10.3)
**2 Affected**	38	20,18	40.0 (±18.9)	21, 17, 0	27.4 (±15.1)
**Unknown**	67	32, 35	51.8 (±17.7)	19, 48	34.5 (±17.7)

Supplementary Table 3

Comparison of linear models performed in this study with and without inclusion of ‘subfamily membership’ as a fixed effect.

	SCZ:PRS (analysis as in paper)				SCZ:PRS (incl. subfamily)			
	Beta	SE	Z-ratio	p-value	Beta	SE	Z-ratio	p-value
**PRS & Affection Status**								
Affected vs Unaffected	0.069	0.032	2.117	0.035	0.059	0.034	1.172	0.088
Psychosis vs Unaffected	0.029	0.023	1.295	0.197	0.019	0.024	0.801	0.425
**PRS & Offspring**								
2 affected vs 0 affected parents	0.064	0.083	0.769	0.446	0.028	0.127	0.216	0.83
Fam affected vs 0 affected parents	0.076	0.061	1.252	0.215	0.071	0.07	1.014	0.317
MI affected vs 0 affected parents	0.209	0.064	3.288	0.002	0.308	0.117	2.634	0.0151
**PRS & Generations**								
G3, G4, G5, G6	0.131	0.049	2.668	0.008	0.089	0.05	1.79	0.075
**PRS & Age at Onset**								
AAO vs PRS	2.093	2.374	0.882	0.381	0.7	2.693	0.26	0.796
**rs1862975 TT genotypes & PRS**								
Affecteds	0.02	0.111	0.181	0.857	-0.108	0.115	-0.943	0.35
Unaffecteds	-0.01	0.038	-0.257	0.797	-0.031	0.04	-0.76	0.449

	MDD:PRS (analysis as in paper)				MDD:PRS (incl. subfamily)			
	Beta	SE	Z-ratio	p-value	Beta	SE	Z-ratio	p-value
**PRS & Affection Status**								
Affected vs Unaffected	-0.0201	0.034	-0.597	0.551	-0.029	0.036	-0.807	0.421
Psychosis vs Unaffected	-0.004	0.024	-0.16	0.873	-0.012	0.025	-0.466	0.641
**PRS & Offspring**								
2 affected vs 0 affected parents	0.076	0.075	1.02	0.313	0.088	0.114	0.768	0.449
Fam affected vs 0 affected parents	0.093	0.063	1.491	0.141	0.089	0.076	1.164	0.25
MI affected vs 0 affected parents	0.081	0.074	1.101	0.277	0.188	0.09	2.089	0.048
**PRS & Generations**								
G3, G4, G5, G6	0.07	0.045	1.548	0.123	0.059	0.047	1.251	0.212
**PRS & Age at Onset**								
AAO vs PRS	0.113	1.866	0.061	0.952	0.595	2.099	0.286	0.778
**rs1862975 TT genotypes & PRS**								
Affecteds	0.097	0.086	1.123	0.265	0.097	0.09	1.071	0.289
Unaffecteds	-0.016	0.037	-0.423	0.673	-0.013	0.04	-0.313	0.755

	BPD:PRS (analysis as in paper)				BPD:PRS (incl. subfamily)			
	Beta	SE	Z-ratio	p-value	Beta	SE	Z-ratio	p-value
**PRS & Affection Status**								
Affected vs Unaffected	0.094	0.03	3.123	0.002	0.066	0.034	1.949	0.053
Psychosis vs Unaffected	0.033	0.021	1.551	0.123	0.01	0.024	0.428	0.669
**PRS & Offspring**								
2 affected vs 0 affected parents	0.141	0.071	1.977	0.05	0.134	0.088	1.527	0.138
Fam affected vs 0 affected parents	0.054	0.061	0.89	0.377	0.01	0.076	0.133	0.895
MI affected vs 0 affected parents	0.172	0.066	2.613	0.013	0.069	0.092	0.756	0.457
**PRS & Generations**								
G3, G4, G5, G6	0.033	0.044	0.747	0.457	0.012	0.045	0.261	0.795
**PRS & Age at Onset**								
AAO vs PRS	-1.563	1.898	-0.823	0.414	-3.63	2.031	-1.787	0.08
**rs1862975 TT genotypes & PRS**								
Affecteds	-0.027	0.091	-0.03	0.765	-0.01	0.095	-0.101	0.92
Unaffecteds	0.019	0.035	0.531	0.596	0.025	0.038	0.656	0.513

Supplementary Table 4

List of tests performed. We have performed 42 tests setting a conservative Bonferroni correction at $P < 0.05/42 = 0.001$.

	Covariates	Random effects	Notes
**PRS & Affection Status (2x3 tests)**			
Affected vs Unaffected	Age, Gender	genetic relatedness	
Psychosis vs Unaffected	Age, Gender	genetic relatedness	
**PRS & Marriedins (1x3 tests)**			
Unaffected MI vs BRA controls	Age, Gender		
**PRS & Offspring (3x3 tests)**			
2 affected vs 0 affected parents	Generation	genetic relatedness	Mean of siblings per parent pair when >1 offspring
Fam affected vs 0 affected parents	Generation	genetic relatedness	Mean of siblings per parent pair when >1 offspring
MI affected vs 0 affected parents	Generation	genetic relatedness	Mean of siblings per parent pair when >1 offspring
**PRS & Generations (1x3 tests)**			
G3, G4, G5, G6	Gender	genetic relatedness	Family members only
**PRS & Age at Onset (1x3 test)**			
AAO vs PRS	Age, Gender	genetic relatedness	
**PRS & Spouse pairs (2x3 tests)**			
All spousepairs	Age, Gender		
Concordant only	Age, Gender		
**Linkage region TDT test (1 test)**			
All available data			Combination of Affymetrix and Illumina data
**rs1862975 TT genotypes & Generations (2 tests)**			
Affecteds	Gender	genetic relatedness	Family members only
Unaffecteds	Gender	genetic relatedness	Family members only
**rs1862975 TT genotypes & PRS (2x3 tests)**			
Affecteds	Age, Gender	genetic relatedness	
Unaffecteds	Age, Gender	genetic relatedness	
**Phenotypic (3 tests)**			
Prevalence married-ins vs population			tested in pedigree irrespective of having genotypes available
Age at onset vs Generation	Gender		tested in pedigree irrespective of having genotypes available
Number of offspring vs Generation	Affection status, PRS		tested in pedigree irrespective of having genotypes available

SUPPLEMENTARY METHODS

Supplementary Methods 1: Linear mixed model as performed in this study.

```
model1a <- asreml(fixed= affection_status ~ 1 + Gender + Age +  zPRS,
                  random= ~giv(kinship matrix),
                  ginverse=list(kinship matrix=Ginv),
                  data= bbfdata,
                  na.method.X="omit", na.method.Y="omit")

wald.asreml(model1a, ssType="conditional", denDF="numeric")
```
